# Preparation, Physicochemical Characterization, Antimicrobial Effects, Biocompatibility and Cytotoxicity of Co-Loaded Meropenem and Vancomycin in Mesoporous Silica Nanoparticles

**DOI:** 10.3390/biomedicines11113075

**Published:** 2023-11-16

**Authors:** Mina Yekani, Robab Azargun, Simin Sharifi, Javid Sadri Nahand, Alka Hasani, Hadi Ghanbari, Zahra Sadat Seyyedi, Mohammad Yousef Memar, Solmaz Maleki Dizaj

**Affiliations:** 1 Infectious and Tropical Diseases Research Center, Tabriz University of Medical Sciences, Tabriz 51548-53431, Iran; 2 Department of Microbiology, Faculty of Medicine, Kashan University of Medical Sciences, Kashan 87137-83976, Iran; 3 Student Research Committee, Kashan University of Medical Sciences, Kashan 87137-83976, Iran; 4 Medicinal Plants Research Center, Maragheh University of Medical Science, Maragheh 55158-78151, Iran; 5 Dental and Periodontal Research Center, Tabriz University of Medical Sciences, Tabriz 51548-53431, Iran; sharifi.ghazi@gmail.com; 6 Immunology Research Center, Tabriz University of Medical Sciences, Tabriz 51548-53431, Iran; 7 Drug Applied Research Center, Tabriz University of Medical Sciences, Tabriz 51548-53431, Iran; 8 Department of Pharmacognosy, Faculty of Pharmacy, Tabriz University of Medical Sciences, Tabriz 51548-53431, Iran; hadighanbari88@gmail.com

**Keywords:** co-delivery, methicillin-resistant *Staphylococcus aureus*, mesoporous silica nanoparticles, vancomycin, meropenem

## Abstract

Mesoporous silica nanoparticles (MSNPs) have been reported as an effective system to co-deliver a variety of different agents to enhance efficiency and improve biocompatibility. This study was aimed at the preparation, physicochemical characterization, antimicrobial effects, biocompatibility, and cytotoxicity of vancomycin and meropenem co-loaded in the mesoporous silica nanoparticles (Van/Mrp-MSNPs). The prepared nanoparticles were explored for their physicochemical features, antibacterial and antibiofilm effects, biocompatibility, and cytotoxicity. The minimum inhibitory concentrations (MICs) of the Van/Mrp-MSNPs (0.12–1 µg/mL) against *Staphylococcus aureus* isolates were observed to be lower than those of the same concentrations of vancomycin and meropenem. The minimum biofilm inhibitory concentration (MBIC) range of the Van/Mrp-MSNPs was 8–64 μg/mL, which was lower than the meropenem and vancomycin MBICs. The bacterial adherence was not significantly decreased upon exposure to levels lower than the MICs of the MSNPs and Van/Mrp-MSNPs. The viability of NIH/3T3 cells treated with serial concentrations of the MSNPs and Van/Mrp-MSNPs were 73–88% and 74–90%, respectively. The Van/Mrp-MSNPs displayed considerable inhibitory effects against MRSA, favorable biocompatibility, and low cytotoxicity. The Van/Mrp-MSNPs could be a potential system for the treatment of infections.

## 1. Introduction

The decreasing susceptibility of microbial pathogens to antimicrobial drugs has resulted in a need for alternative antimicrobial therapy. Antibiotic combination therapy may be an alternative option against antibiotic-resistant bacteri1a or for decreasing the emergence of resistant strains [1]. Vancomycin is a glycopeptide antimicrobial agent applied in the antibiotic therapy of Gram-positive bacteria-caused infections because it suppresses the synthesis of cell wall peptidoglycan. That the vancomycin molecule is large may be a reason for its inactivity against Gram-negative bacteria [2]. *Staphylococcus aureus* is commonly isolated from healthcare infections with high rates of fatality. *S. aureus* is commonly responsible for surgical site infections, wound infections, sepsis, pneumonia, bone infections, and endocarditis [3]. An important clinical challenge related to nosocomial infections caused by this pathogen is its non-sensitivity to different groups of antibacterial drugs which limit the therapeutic options for its infections [4]. Several studies have indicated that in polymicrobial infections with Gram-negative pathogens, vancomycin does not show identical effects against methicillin-resistant *S. aureus* (MRSA) [5,6]. To increase the spectrum of antibacterial effects as well as to decrease vancomycin’s side effects, the combination of it with various antimicrobial agents may be applicable. Vancomycin has indicated a synergistic antibacterial effect with carbapenems against MRSA [7]. Carbapenems are the primary choice for the antimicrobial therapy of multidrug-resistant (MDR) Gram-negative pathogens. The combination of vancomycin and carbapenem has an improved antibacterial property against MRSA [6,7]. The combinatory treatments of carbapenem (meropenem) with vancomycin are commonly used for the primary treatment of nosocomial infections, because they offer a noticeably high and increased antimicrobial spectra against Gram-positive and Gram-negative bacteria [8]. The use of the co-delivery system of carbapenem and vancomycin may be a potential way to treat MRSA. Nanomaterials have many applications in biomedicine, including drug delivery, which have been investigated for the treatment of various diseases [9,10]. 

Among various drug delivery approaches, mesoporous silica nanoparticles (MSNPs) are a desirable carrier for loading biologically active compounds due to their favorable physicochemical properties and biocompatibility [11]. 

A variety of biologically active compounds, including chemotherapeutic agents, antimicrobial agents, polymers, fluorescent dyes, and therapeutic peptides, have been loaded on the MSNPs for controlled release [12].

In addition, MSNPs have been investigated to co-deliver a variety of different agents to enhance efficiency and improve biocompatibility [13]. The antimicrobial properties and biocompatibility of meropenem-loaded MSNPs and vancomycin-loaded MSNPs have been studied previously. It has been shown that the loading of these antibiotics on the MSNPs will not lead to a decrease in their antimicrobial effects and has not been associated with unfavorable effects on its biocompatibility properties [14,15,16]. If a drug delivery system can be designed for the co-loading of vancomycin and meropenem, in addition to the possibility of increasing the antimicrobial effects by inducing synergistic effects, the antimicrobial spectrum will be increased due to the simultaneous presence of the drugs. In this study, vancomycin and meropenem were co-loaded on MSNPs (Van/Mrp-MSNPs). Then, the antimicrobial effects of the Van/Mrp-MSNPs against MRSA and their biocompatibility and cytotoxicity were determined in in vitro conditions. 

## 2. Material and Methods 

### 2.1. Preparation of Vancomycin/Meropenem-Loaded MSNPs

For loading vancomycin and meropenem on the MSNPs, 2 mg of MSNPs was added to 0.5 mL of vancomycin (2 mg/mL) and 0.5 mL of meropenem (2 mg/mL) and stirred gently at room temperature for 24 h. Free molecules of antibiotics were removed by centrifuging and washing with deionized water. For studying antibiotic loading efficiency, Van/Mrp-MSNPs (100 mg) were dissolved in EDTA (0.5 M, pH 7.5) under stirring for 45 min. Then, 1 mL of aqueous section was applied to evaluate the drug-loading efficacy. The optical density (OD) of the suspension was measured at 280 nm and 297 nm for vancomycin and meropenem, respectively by a UV-visible spectrophotometer. 

### 2.2. Characterization

#### 2.2.1. Fourier-Transform Infrared Spectroscopy (FTIR) 

The functional groups of vancomycin, meropenem, the mesoporous silica nanoparticles, and Van/Mrp-MSNPs were studied using FTIR by means of a spectrophotometer in the range of 4000–400 cm^−1^, using potassium bromide powder as the standard compound.

#### 2.2.2. Dynamic Light Scattering (DLS) 

The size and the charge (Zeta potential) were determined using the DLS method (DLS, Malvern, Cambridge, Massachusetts, UK). For this, Van/Mrp-MSNPs were suspended in 1 mL of distilled water and transferred to a test tube of the DLS approach. 

#### 2.2.3. Scanning Electron Microscope (SEM)

The SEM was used to determine of morphology and structure of the prepared nanoparticles.

### 2.3. Drug Loading and the Drug Release Pattern 

Entrapment loading efficiency and drug loading amount of Van/Mrp-MSNPs were determined by the calibration standard (10–50 µg/mL) according to the following formulas [17,18]:

$$\mathrm{Drug}\;\mathrm{loading}\;\mathrm{amount}\;(\%)=\frac{\mathrm{drug}\;\mathrm{content}}{\mathrm{Total}\;\mathrm{amount}\;\mathrm{of}\;\mathrm{drug}\;\mathrm{loaded}\;\mathrm{particles}}\times 100\%$$

$$\mathrm{Entrapment}\;\mathrm{loading}\;\mathrm{efficiency}\;(\%)=\frac{\mathrm{drug}\;\mathrm{content}}{\mathrm{total}\;\mathrm{drug}\;\mathrm{added}}\times 100\%$$

### 2.4. Drug Release Pattern

Release patterns of meropenem and vancomycin from MSNPs were determined using drug dissolution device no. 2. First, PBS (100 mL) was transferred to each well of the device. Then, 5 mg of the tested Van/Mrp-MSNPs were added to all wells. The pH, temperature, and stirrer speed were adjusted to 7.4, at 37 °C, and to 100 rpm, respectively. The absorbance of specimens was determined using a UV spectrophotometer (Shimadzu, Kyoto, Japan). An ultraviolet (UV) spectrophotometer was used, with one mL of the nanoparticle solution and the adsorption number adjusting the Landa Max at a wavelength of 280 nm for vancomycin and 297 nm for meropenem, respectively.

### 2.5. Bacterial Isolates

Five non-duplicated biofilm-producing MRSA isolates recovered from clinical specimens during 2020 from Tabriz, Iran, and *S. aureus* ATCC 43300 were selected to determine microbial assays in the current study. The bacteria were identified by the standard microbiological procedures [19]. The MRSA isolates were primarily screened by their susceptibility pattern to a cefoxitin disk (30 µg), using the disk diffusion method on Müller–Hinton agar [20]. The biofilm-forming ability of isolates was studied according to the previously demonstrated microtiter plate test (MPT) [21].

### 2.6. Antibacterial Effects 

The broth microdilution technique was used to determine the bacterial inhibitory effects of vancomycin, meropenem, MSNPs, and Van/Mrp-MSNPs using the CLSI guideline [22]. The antibacterial activity of the antibiotic’s combination was determined using the checkerboard method. For the checkerboard method, the MIC of drugs was firstly determined alone by broth microdilution and then in combination against bacterial isolates in a 96-well microplate according to the previously described method [1]. The wells without antimicrobial agents were considered as the growth control of bacteria. The FIC indexes were determined and interpreted using breakpoints presented in previous reports [1]. 

### 2.7. Antibiofilm Property 

Biofilm inhibitory properties of Van/Mrp-MSNPs, meropenem, and vancomycin were assayed by determination of minimum biofilm inhibitory concentration (MBIC). Firstly, the microbial suspension was prepared in TSB (0.5 McFarland) and 100 µL of it was transferred to the wells of a microplate containing 100 µL of TSB supplemented with 0.25% glucose and incubated at 36 °C. Then, free antibiotics and prepared Van/Mrp-MSNPs at serial amounts were transferred to wells after 24h, and the incubating procedure was performed at 36 °C. The contents of microplates were removed after 20 h and wells were washed using PBS and 200 µL of fresh TSB was added to the wells. The OD_650 nm_ of microplates was detected before and after incubation at 36 °C for 8 h. The MBIC of a compound was defined as the lowest level associated with an OD_650 nm_ difference at or below 10% of the mean of two standard wells OD. 

### 2.8. The Effect of Van/Mrp-MSNPs on the Microbial Attachment

To evaluate the inhibitory effect of Van-MSNPs on the adhesion of bacteria to surfaces, sub-inhibitory concentrations (1/2 MIC) of Van/Mrp-MSNPs were transferred to the wells of a microplate. Microbial suspensions (0.5 McFarland) diluted in trypticase soy broth (TSB) (1:10) with and without Van/Mrp-MSNPs were incubated at 36 °C for 20 h. After incubation, the well content was removed and washed with PBS. The formed biofilm in the well was studied using MPT assay [23]. 

### 2.9. Van/Mrp-MSNPs Effects on RBCs

The hemocompatibility of Van/Mrp-MSNPs was investigated by determining the lytic property of red blood cells (RBCs) and its effects on the human erythrocyte sedimentation rate (ESR). For isolating RBCs, the blood sample was centrifuged for 5 min at 4000 rpm and sediment was washed three times with PBS (pH: 7.4) for 10 min. Then, 1 mL diluted cells in PBS (1:10) were treated using different levels of Van/Mrp-MSNPs at 37 °C for 6 h. All tubes were centrifuged at 4000 rpm for 10 min and 200 μL of each tube’s supernatant was transferred into the wells of a microplate. The OD of released hemoglobin from RBCs upon exposure to Van/Mrp-MSNPs, water (positive controls of hemolysis), and PBS (negative controls of hemolysis) was determined at 540 nm for calculating hemolysis rates. The rate of hemolysis was determined by the below equation:

$$\mathrm{Hemolysis}\;\mathrm{Rate}\;(\%)=\frac{\mathrm{OD}\left(s\right)-\;\mathrm{OD}\left(-\right)}{\mathrm{OD}\left(+\right)-\;\mathrm{OD}\left(-\right)}\times 100$$

As OD (+) is the absorbance of the sample upon exposure to water, OD(s) is the absorbance of the tested specimens in the presence of various amounts of Van/Mrp-MSNPs, and OD(−) is the absorbance of the PBS-treated sample without nanoparticles [24]. Optical microscopy was used to study the morphology of RBC at the magnification of ×40. The effect of Van/Mrp-MSNPs on ESR was investigated by adding the Van/Mrp-MSNPs to total blood in a western green tube comprising 3.2% sodium citrate at room temperature for 1h. The tube containing PBS (pH 7.4) and the blood sample was considered the control. The RBC column was measured as the ESR in mm/h [25].

### 2.10. Interaction of Van/Mrp-MSNPs and Human Plasma Protein 

The interaction of Van/Mrp-MSNPs and human plasma protein was studied according to the previously described method [24]. It was evaluated by treating plasma with Van/Mrp-MSNPs and sodium dodecyl-sulfate polyacrylamide gel electrophoresis (SDS-PAGE) method for separating proteins based on their size. Briefly, 200 μL of human blood plasma was treated with Van/Mrp-MSNPs added to PBS (pH 7.4) for 4 h at 37 °C. Free proteins on the surface of Van/Mrp-MSNPs were removed by washing one to three times using 2 mL PBS (pH 7.4). Three concentrations of human plasma without exposure to Van/Mrp-MSNPs were considered as the control. The plasma after treatment with Van/Mrp-MSNPs was loaded on a 12% polyacrylamide gel for separation by electrophoresis in the presence of protein size marker. Coomassie brilliant blue R-250 (0.25%) and destaining agent (50 mL of methanol, 40 mL of water, and 10 mL of acetic acid) were used for staining and destaining the gel, respectively. The gel photography was performed by a high-resolution scanner. The bands were studied in reference to a size marker to detect the molecular weight of the proteins interacting with Van/Mrp-MSNPs [24]. 

### 2.11. Cytotoxic Effects of Van/Mrp-MSNPs In Vitro

Van/Mrp-MSNPs cell toxicity against NIH/3T3 was determined using the MTT assay. In summary, NIH/3T3 cells were transferred into the 96-well microplate (10^4^ cells/well) for 24 h at 37 °C. Then, the medium was replaced with 100 µL/well of serial concentration (8–256 μg/mL) of Van/Mrp-MSNPs in an antibiotic-free RPMI medium. The microplate was incubated at 37 °C for 24 h. The well containing NIH/3T3 without treatment by Van/Mrp-MSNPs and empty wells were considered as the negative control and blank, respectively. Then, 20 μL of MTT was added to the well at a concentration of 0.5 mg/mL and the microplate was incubated at 37 °C for 4 h. The MTT was reduced to a formazan product by living cells. The free MTT was removed from wells and 200 μL/well of DMSO was added to wells to dissolve the formazan crystals. The OD of wells was determined at 570 nm in triplicate and viability of NIH/3T3 was calculated using the previously mentioned equation [24].

### 2.12. Statistical Analysis

The data analyses were performed using SPSS software version 18. The evaluation of the results among different groups was performed using non-parametric examinations and *p* values of ≤0.05 were defined as significant. 

## 3. Results 

**Figure 1:**
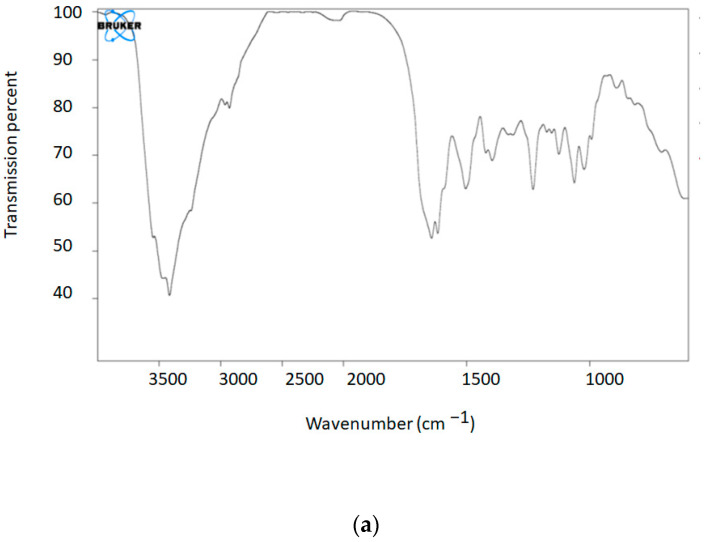

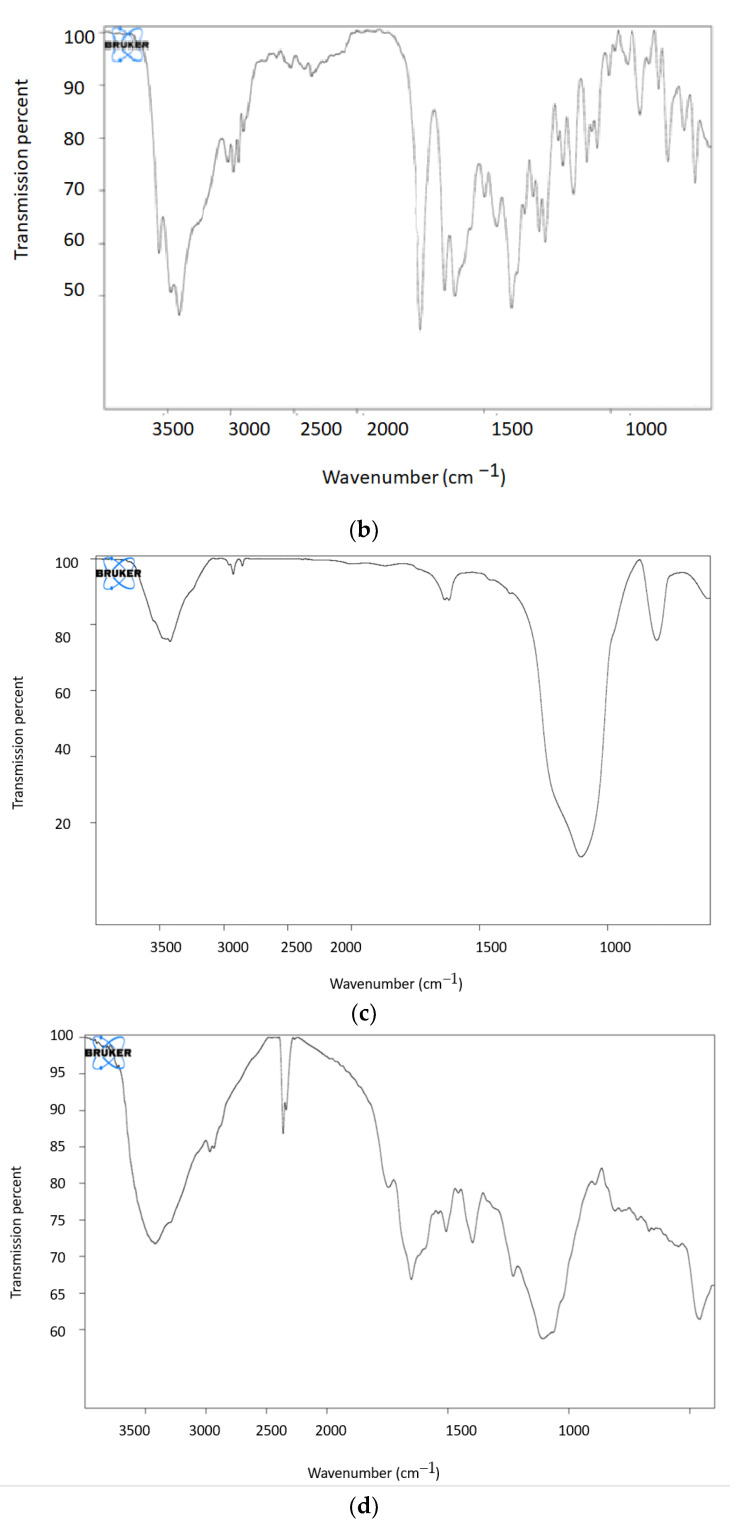
FTIR peaks of functional groups of vancomycin (**a**), meropenem (**b**), the MSNPs (**c**) and Van/Mrp-MSNPs (**d**).

In Figure 1a–d, the functional groups of vancomycin, meropenem, the mesoporous silica nanoparticles, and Van/Mrp-MSNPs are shown in the FTIR peaks. All the functional groups are represented as FTIR peaks. All the characteristic peaks of the drugs are also in the FTIR spectrum of the Van/Mrp-MSNPs.

**Figure 2:**
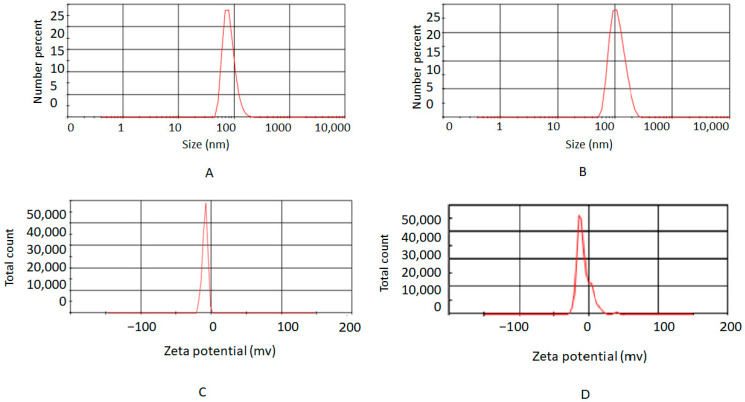
Particle size and the zeta-potential: (**A**) particle size distribution of free MSNPs, (**B**) particle size distribution of Van/Mrp-MSNPs, (**C**) zeta-potential of free MSNPs, and (**D**) zeta-potential of Van/Mrp-MSNPs.

The particle size of the MSNPs and Van/Mrp-MSNPs is presented in Figure 2. According to the DLS results, the diameters of the nanoparticles were 81.52 nm for the MSNPs and 92.81 nm for the Van/Mrp-MSNPs. The zeta-potential of the MSNPs and Van/Mrp-MSNPs were −9 mV and −11 mV, respectively (Figure 2). In this study, the drug loading and entrapment efficiencies of meropenem were 15.1% and 31.7%, and for vancomycin, they were 17.25 and 35.4% with the MSNPs, respectively. 

**Figure 3:**
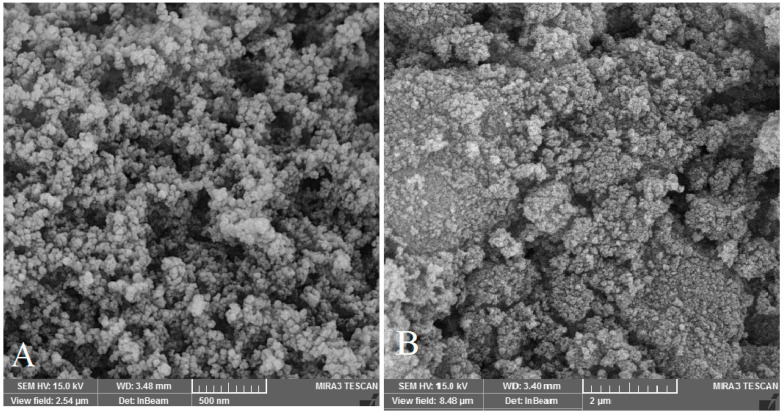
The morphology of free MSNPs (**A**) and Van/Mrp-MSNPs (**B**).

**Figure 4:**
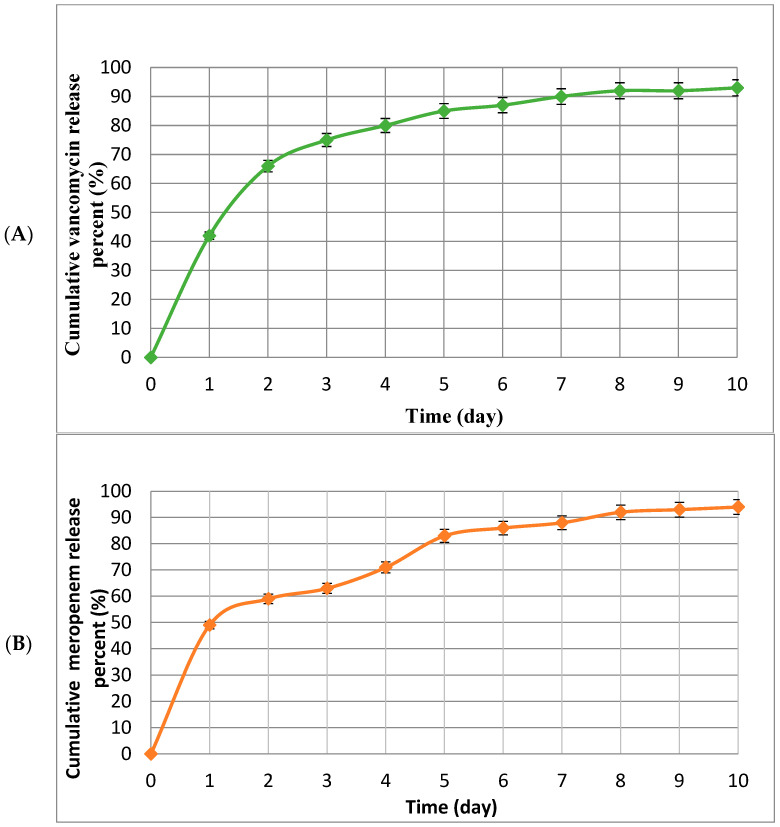
The pattern of vancomycin (**A**) and meropenem (**B**) release from Van/Mrp-MSNPs.

According to the drug release pattern, the Van/Mrp-MSNPs exhibited a nearly 50% meropenem release during the first day, and a lower concentration during the following days (10%); and showed a 40% release of vancomycin during the first 2h and decreased amounts during the following days (30%) (Figure 3 and Figure 4). In addition, both antibiotics indicated a sustained release pattern until the 10th day. 

### 3.1. Antibacterial Effects

According to the results of the microbroth dilution method, the MICs of vancomycin and meropenem against the *S. aureus* isolates were 0.5–2 µg/mL and 64–128 µg/mL, respectively (Table 1). A noticeable inhibitory effect of the free MSNPs was not observed against the bacterial isolates. Antimicrobial synergic effects (FICI ≥ 0.5) were observed with the combination of vancomycin/meropenem against *S. aureus* (five of six isolates) (Table 1).

**Figure 5:**
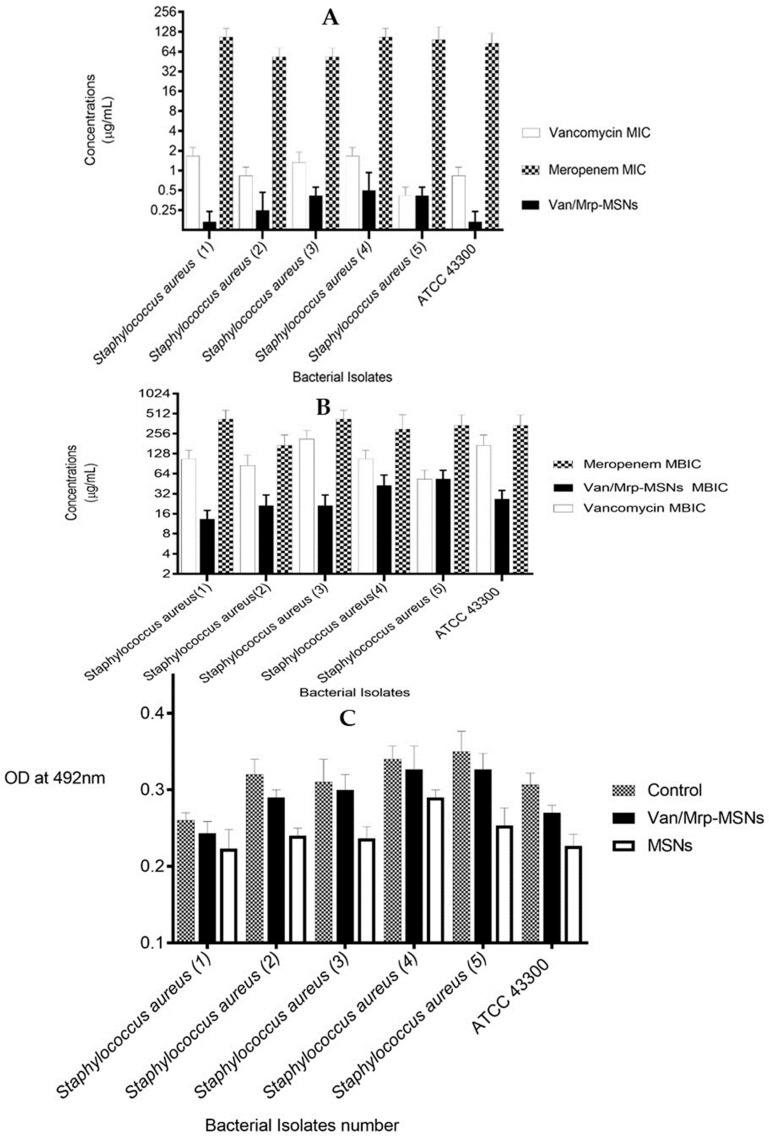
(**A**) The inhibitory levels of free vancomycin, meropenem, and Van/Mrp-MSNPs on tested microorganisms, MIC: minimum inhibitory concentration, (**B**) The antibiofilm effects of vancomycin, meropenem, and Van/Mrp-MSNPs, MBIC: minimum biofilm inhibitory concentration, (**C**) effects of Van/Mrp-MSNPs and MSNPs on the microbial adherence to surfaces.

The Van/Mrp-MSNPs had an inhibitory effect in all the examined bacterial isolates. The Van/Mrp-MSNPs inhibited the growth of bacteria at a lower concentration (0.12–1 µg/mL) than vancomycin (0.5–2 µg/mL) and meropenem (64–128 µg/mL) did (Figure 5A). 

### 3.2. Antibiofilm Effects 

Both vancomycin and meropenem showed considerable biofilm inhibitory effects at higher levels than the MICs. The MBICs of the Van/Mrp-MSNPs were more than the concentration required for inhibitory effects on the planktonic growth (MICs) against both *S. aureus* isolates. The MBIC range of the Van/Mrp-MSNPs was 8–64 μg/mL, which was lower than the MBICs of meropenem (128–512 μg/mL) and vancomycin (32–256 μg/mL) (Figure 5B). The microbial cell adherence was evaluated to study the effect of the sub-MICs levels (0.5 MIC) of the Van/Mrp-MSNPs on the attachment of bacteria to the well of polyester microplate wells. The bacterial adherence was not significantly decreased after the treatment with the MSNPs and Van/Mrp-MSNPs than the control wells (containing bacterial suspension without antimicrobial agent) (Figure 5C). 

### 3.3. Hemolytic Effects and RBCs Sedimentation upon Exposure to Van/Mrp-MSNPs

**Figure 6:**
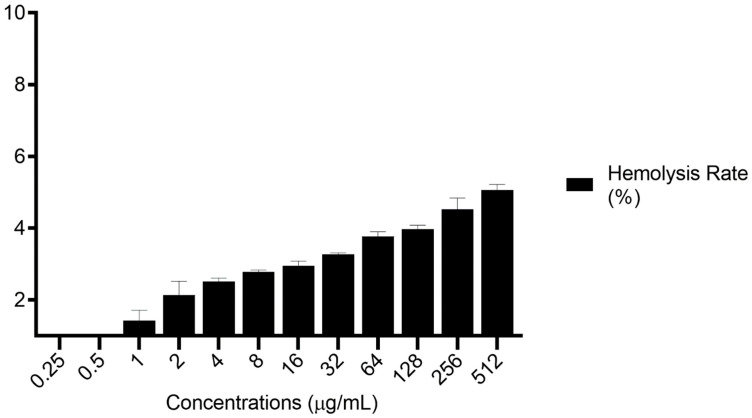
Hemolytic effects of Van/Mrp-MSNPs at serial concentrations of 0.25 to 512 μg/mL.

To study the hemolytic effects of Van/Mrp-MSNPs, RBCs were exposed to different concentrations (0.25 to 512 µg/mL) of the Van/Mrp-MSNPs for 4 h. As is presented in Figure 6, the lysis rates of the RBCs were increased in a dose-dependent manner. The examination by optical microscopy confirmed that the Van/Mrp-MSNPs do not exhibit a detectable change in the morphology of RBCs. The ability of the Van/Mrp-MSNPs to aggregate the RBCs was studied by treating the RBCs with the Van/Mrp-MSNPs. The ESR was not affected in the presence of 512 μg/mL of the Van/Mrp-MSNPs differently to with the negative control.

### 3.4. Interaction of Van/Mrp-MSNPs and Human Plasma Protein 

**Figure 7:**
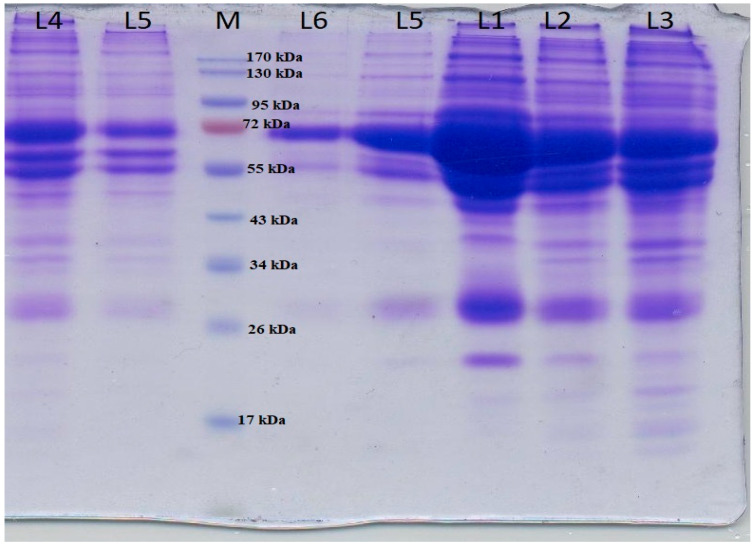
SDS-PAGE result. Interaction of plasma proteins with Van/Mrp-MSNPs was revealed after one (lane 4), two (lane 5), and three (lane 6) washing steps The bands of plasma proteins after 20 (lane 2), 10 (lane 3), and 5 (lane 1) dilutions.

In the present study, SDS-PAGE was used for the separation of the hard protein corona of human blood plasma treated with the Van/Mrp-MSNPs after washing three times with 1.5 mL PBS (Figure 7, L4–L6). The interaction of the Van/Mrp-MSNPs and proteins was investigated by treating the different dilutions of plasma with the Van/Mrp-MSNPs as the control (Figure 7, L1–L3). The SDS-PAGE exhibited a significant band with a molecular weight of 66.5 kDa that may show the interaction of nanoparticles with albumin. 

### 3.5. Cytotoxic Effects of Van/Mrp-MSNPs 

**Figure 8:**
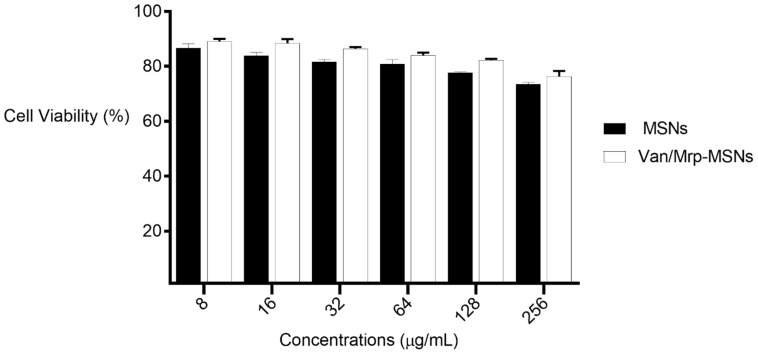
NIH/3T3 cell viability upon exposure to serial concentrations of Van/Mrp-MSNPs and MSNPs.

The in vitro cytotoxicity of the MSNPs and Van/Mrp-MSNPs was assessed on NIH/3T3 (a fibroblast cell line) cells using an MTT assay. The viability of the NIH/3T3 cells treated with serial concentrations of the MSNPs and Van/Mrp-MSNPs was 73–88% and 74–90%, respectively (Figure 8). 

## 4. Discussion

The fast advance in nanomaterial technology has been described in various areas of tissue engineering and biomedicine such as, drug delivery approach, applications in cancer therapy, and antimicrobial agents. Several drug delivery system have been studied to improve the efficiency of antibiotics by increasing the accumulation and pharmacokinetics and reducing the drug’s side effect [14,24]. Theoretically, nanomaterials have a longer half-life in the human body than free antimicrobial drugs, which could be useful for increasing the release of therapeutic agents [14]. MSNPs are an appropriate drug delivery system due to their desirable properties such as desirable surface area, variable pore diameter, stability, biocompatibility, cost effectiveness, and ease of synthesis on a great scale [14]. MSNPs can be the carrier for different antimicrobial drugs against different microorganisms [14,15]. MSNPs have also been reported as potential carriers for co-delivery and combination therapy against cancer [26]. In the current in vitro investigation, MSNPs have been studied as a vehicle for the co-delivery of vancomycin and meropenem combination therapy against resistant bacteria. The diameters of the Van/Mrp-MSNPs and MSNPs were 81.52 nm and 92.81 nm, respectively and the zeta-potential of the MSNPs and Van/Mrp-MSNPs were −9 mV and −11 mV, respectively. The zeta-potential provides important information about the surface charge of nanoparticles, which may be helpful to indicate the stability and the interaction of a particle with biological structures. Negative zeta-values have been reported to be effective in interaction with cellular targets and enhance the biological activities of nanoparticles. Two critical parameters in preparing the delivery system are the drug loading and entrapment efficiencies, which were 15.1% and 31.7% for meropenem and for vancomycin were 17.25 and 35.4% in the MSNPs, respectively. The different values of loading efficiencies have been reported for several antimicrobial agents in MSNPs such as meropenem (18.4%) [14], polymyxin B (11–37%) [27], and tetracycline (11.7–18.7%) [28]. The Van/Mrp-MSNPs exhibited the release of nearly 50% of the meropenem during the first day and a 40% release of the vancomycin during the first day and a sustained release pattern until the 10th day. It has been proposed that the release of the loaded antibiotics during the primary hours causes a quick releasing pattern and the stronger electrostatic bond among the drugs and nanoparticles leads to the sustained patterns of drug release until the 12th day [14,25]. MSNPs with high loading levels of antimicrobial agents (but with weak bonds between antibiotics and nanoparticles) have not been indicated to have a desirable interaction with the bacterial cell structure. However, MSNPs with a lower but stronger bond between antimicrobial agents and MSNPs have shown potential to bond to the membrane of bacteria due to a slow-release pattern. The result of the checkerboard exhibited antimicrobial synergic effects (FICI ≥ 0.5) for the combination of vancomycin/meropenem against *S. aureus* (five of six isolates). The in vitro synergic effects of the meropenem and vancomycin combination have been reported against *S. aureus* [7]. The resistance of bacteria in biofilm form has been attributed to several mechanisms: the matrix of the biofilm, which forms a barrier which decreases the antibiotic’s penetration, and the existence of slow-growing microorganisms with low metabolic rates [21]. The MBICs of the Van/Mrp-MSNPs were more than the concentration required for inhibitory effects on the planktonic growth (MICs) against both *S. aureus* isolates. A higher MBIC than MIC of meropenem-loaded MSNPs has been reported against biofilm-forming carbapenem-resistant *Pseudomonas aeruginosa* [15]. MSNPs have also been indicated to be effective drug carriers in the delivery of other drugs, including levofloxacin and lysozyme, into biofilms [29]. The ability of MSNPs’ coatings to release the new cephalosporin known as Zinforo has been shown to have a significant effect on the primary step of *Escherichia coli* biofilm formation [30]. Adherence is the essential step for establishing microbial cell colonization on the host tissue surfaces. Therefore, anti-adhesion procedures can be an applicable way to prevent or treat infections. Bacterial adherence was not significantly decreased after the treatment with the MSNPs and Van/Mrp-MSNPs compared to the controls. According to these results, the Van/Mrp-MSNPs can inhibit preformed biofilms by a mechanism other than inhibitory effects on the initial steps of bacterial adherence. However, more detailed studies are needed to detect its biofilm inhibitory mechanism.

The usage of nanomaterials as a drug delivery approach may be associated with undesirable effects on the host tissue and cells. Moreover, biocompatibility with host cells is important to understand the route of administration of a compound for treatment. The compatibility with blood cells is an essential in vitro and in vivo feature of nanomaterials. The hemolytic properties of nanocarriers may be triggered due to several mechanisms such as an alternation in the enzymatic effects and increased susceptibility to oxidative and osmotic stress. The hemolytic activity is an essential property for the in vitro study of the biocompatibility of drug delivery systems [24]. The concentration of MSNPs has been reported to be effective on the rate of RBC lysis. [14,31]. The significant hemolysis effect of MSNPs has been reported to extend up to about 14.2% at the level of 500 µg/mL [32]. This hemolytic property has been reported to be related to the presence of silanol groups on the MSNPs surface which induce oxidative stress due to the promotion of reactive oxygen species (ROS) formation. In addition, MSNPs possess a considerable affinity to tetra-alkyl ammonium motifs on the surface of RBCs and can bind electrostatically with cell membrane peptides. The results of the present study indicated that the effects of the Van/Mrp-MSNPs at low concentrations on the RBCs are insignificant in an in vitro system. According to the international guidelines, compounds can meet the blood safety necessity in clinical usages only if the hemolysis levels are lower than 5% [33].

The interactions with cell surfaces and plasma proteins has significant effects on the biocompatibility and biodistribution of nanomaterials [24]. The intravenous usage of nanoparticles may trigger and/or suppress immunological reactions through effects on the blood cells and proteins. The interaction between a nanoparticle with plasma proteins is associated with forming an active particle–protein corona that plays an essential biological function in nanoparticles including cellular uptake, inducing immune response, toxicity, and clearance [34]. Albumins in general are transport proteins that bind to different compounds and play an important role in transferring them. Some studies have shown the interaction of MSNPs with albumin in serum [35]. The connection of a drug or molecule with proteins in plasma can meaningfully impact the cellular uptake and transfer in the host circulating system and body environments. Albumin is one of the principal compounds in the human plasma and plays an important role in regulating blood pressure and the transfer of drug molecules in the blood. The physicochemical characterization of the nanoparticles significantly influences the interaction of a drug delivery system with the cell and plasma proteins [36]. For applications of nanomaterials such as in biomedicine, it is required to evaluate their biological safety. The nanoscale size and the large surface area of nanoparticles lead to a higher toxicity in comparison to the substance, which has a micrometer or larger size. 

The viability of the NIH/3T3 cells treated with serial concentrations of MSNPs and Van/Mrp-MSNPs were 73–88% and 74–90%, respectively. According to the ISO 10993-5:2009 guideline, if the in vitro cell viability can be maintained at ≥70% upon the exposure of a certain compound, it can be considered as non-toxic [37]. Several reports have indicated the biosafety of MSNPs and their slow degradation in the body. However, some levels of the in vitro cytotoxic effects of MSNPs have been observed in various cell lines [38]. In vitro toxicity studies have displayed MSNPs which induce their cytotoxicity in a time- and concentration-dependent manner [39,40]. It is well-known that different cell lines can show a different uptake level of MSNPs that significantly affects their cytotoxicity. It has been shown that the MSNP uptake by MCF-7 cells and subsequently its toxic properties were induced in a dose-dependent manner [41]. Whereas, no detectable uptake and toxicity has been observed in the BJ cells after 24 h treatment with MSNPs [41]. The cytotoxicity effect of MSNPs is due to inducing ROS formation and DNA breaks that are pointedly related to the characterization of the surface silanol groups of MSNPs [42]. 

## 5. Conclusions 

MSNPs can be used as a potential system for the co-delivery of antimicrobial agents in treating infections caused by drug-resistant microbial pathogens. According to the results of the present study, Van/Mrp-MSNPs show favorable physicochemical features and low cytotoxicity. The antimicrobial and antibiofilm effects of the Van/Mrp-MSNPs were higher than vancomycin, meropenem, and their combination against MRSA. Therefore, MSNPs could be an applicable potential drug co-delivery system against drug-resistant pathogens. 

## Figures and Tables

**Table 1:** Antimicrobial effects of vancomycin and meropenem in combination against bacterial effects.

Isolate Number	MIC of Vancomycin (µg/mL)		MIC of Meropenem (µg/mL)		FICI
	Alone	In Combination	Alone	In Combination	
SA1	2	0.5	128	32	0.5
SA2	1	0.25	64	8	0.37
SA3	1	0.5	64	8	0.62
SA4	2	0.5	128	32	0.5
SA5	0.5	0.125	128	32	0.5
ATCC 43300	1	0.125	64	16	0.37

## Data Availability

No data was associated in the manuscript.

## References

[1] Horcajada J.P., Montero M., Oliver A., Sorlí L., Luque S., Gómez-Zorrilla S., Benito N., Grau S. (2019). Epidemiology and treatment of multidrug-resistant and extensively drug-resistant Pseudomonas aeruginosa infections. Clin. Microbiol. Rev..

[2] Rybak M.J. (2006). The pharmacokinetic and pharmacodynamic properties of vancomycin. Clin. Infect. Dis..

[3] Wu Q., Sabokroo N., Wang Y., Hashemian M., Karamollahi S., Kouhsari E. (2021). Systematic review and meta-analysis of the epidemiology of vancomycin-resistance Staphylococcus aureus isolates. Antimicrob. Resist. Infect. Control.

[4] Turner N.A., Sharma-Kuinkel B.K., Maskarinec S.A., Eichenberger E.M., Shah P.P., Carugati M., Holland T.L., Fowler V.G. (2019). Methicillin-resistant Staphylococcus aureus: An overview of basic and clinical research. Nat. Rev. Microbiol..

[5] Totsuka K., Shiseki M., Kikuchi K., Matsui Y. (1999). Combined effects of vancomycin and imipenem against methicillin-resistant Staphylococcus aureus (MRSA) in vitro and in vivo. J. Antimicrob. Chemother..

[6] Rochon-Edouard S., Pestel-Caron M., Lemeland J.-F., Caron F. (2000). In vitro synergistic effects of double and triple combinations of β-lactams, vancomycin, and netilmicin against methicillin-resistant Staphylococcus aureus strains. Antimicrob. Agents Chemother..

[7] Mohammadi-Berenjestanaki H., Khori V., Shirzad-Aski H., Ghaemi E.A. (2020). In vitro synergistic effect of vancomycin and some antibacterial agents against clinical methicillin-resistant and sensitive Staphylococcus aureus isolates. Microb. Drug Resist..

[8] Wicha S.G., Kees M.G., Kuss J., Kloft C. (2015). Pharmacodynamic and response surface analysis of linezolid or vancomycin combined with meropenem against Staphylococcus aureus. Pharm. Res..

[9] Yetisgin A.A., Cetinel S., Zuvin M., Kosar A., Kutlu O.J.M. (2020). Therapeutic nanoparticles and their targeted delivery applications. Molecules.

[10] Maleki Dizaj S., Sharifi S., Jahangiri A. (2019). Electrospun nanofibers as versatile platform in antimicrobial delivery: Current state and perspectives. Pharm. Dev. Technol..

[11] Ahmed H., Gomte S.S., Prathyusha E., Prabakaran A., Agrawal M., Alexander A. (2022). Biomedical applications of mesoporous silica nanoparticles as a drug delivery carrier. J. Drug Deliv. Sci. Technol..

[12] Zhuang J., Yu Y., Lu R. (2022). Mesoporous silica nanoparticles as carrier to overcome bacterial drug resistant barriers. Int. J. Pharm..

[13] Paris J.L., Vallet-Regí M. (2020). Mesoporous silica nanoparticles for co-delivery of drugs and nucleic acids in oncology: A review. Pharmaceutics.

[14] Memar M.Y., Yekani M., Ghanbari H., Shahi S., Sharifi S., Maleki Dizaj S. (2020). Biocompatibility, cytotoxicity and antibacterial effects of meropenem-loaded mesoporous silica nanoparticles against carbapenem-resistant Enterobacteriaceae. Artif. Cells Nanomed. Biotechnol..

[15] Memar M.Y., Yekani M., Ghanbari H., Nabizadeh E., Vahed S.Z., Dizaj S.M., Sharifi S. (2021). Antimicrobial and antibiofilm activities of meropenem loaded-mesoporous silica nanoparticles against carbapenem-resistant Pseudomonas aeruginosa. J. Biomater. Appl..

[16] Memar M.Y., Yekani M., Farajnia S., Ghadiri Moghaddam F., Nabizadeh E., Sharifi S., Maleki Dizaj S. (2023). Antibacterial and biofilm-inhibitory effects of vancomycin-loaded mesoporous silica nanoparticles on methicillin-resistant staphylococcus aureus and gram-negative bacteria. Arch. Microbiol..

[17] Hanafi-Bojd M.Y., Ansari L., Mosaffa F., Malaekeh-Nikouei B. (2017). The effect of mesoporous silica nanoparticles loaded with epirubicin on drug-resistant cancer cells. Nanomed. J..

[18] Maleki Dizaj S., Lotfipour F., Barzegar-Jalali M., Zarrintan M.-H., Adibkia K. (2017). Ciprofloxacin HCl-loaded calcium carbonate nanoparticles: Preparation, solid state characterization, and evaluation of antimicrobial effect against Staphylococcus aureus. Artif. Cells Nanomed. Biotechnol..

[19] Tille P. (2015). Bailey & Scott’s Diagnostic Microbiology-E-Book.

[20] In C. (2018). Performance Standards for Antimicrobial Susceptibility Testing.

[21] Sopirala M.M., Mangino J.E., Gebreyes W.A., Biller B., Bannerman T., Balada-Llasat J.M., Preeti Pancholi P. (2010). Synergy testing by Etest, microdilution checkerboard, and time-kill methods for pan-drug-resistant Acinetobacter baumannii. Antimicrob. Agents Chemother..

[22] Weinstein M.P. (2018). Methods for Dilution Antimicrobial Susceptibility Tests for Bacteria That Grow Aerobically.

[23] Hayat S., Muzammil S., Rasool M.H., Nisar Z., Hussain S.Z., Sabri A.N., Jamil S. (2018). In vitro antibiofilm and anti-adhesion effects of magnesium oxide nanoparticles against antibiotic resistant bacteria. Microbiol. Immunol..

[24] Rahimi M., Safa K.D., Salehi R. (2017). Co-delivery of doxorubicin and methotrexate by dendritic chi-tosan-g-mPEG as a magnetic nanocarrier for multi-drug delivery in combination chemotherapy. Polym. Chem..

[25] Achilli C., Grandi S., Ciana A., Guidetti G.F., Malara A., Abbonante V., Cansolino L., Tomasi C., Balduini A., Fagnoni M. (2014). Biocompatibility of functionalized boron phosphate (BPO4) nanoparticles for boron neutron capture therapy (BNCT) application. Nanomed. Nanotechnol. Biol. Med..

[26] Xu P., Yao J., Li Z., Wang M., Zhou L., Zhong G., Zheng Y., Li N., Zhai Z., Yang S. (2020). Therapeutic effect of doxorubicin-chlorin E6-loaded mesoporous silica nanoparticles combined with ultrasound on triple-negative breast cancer. Int. J. Nanomed..

[27] Gounani Z., Asadollahi M.A., Meyer R.L., Arpanaei A. (2018). Loading of polymyxin B onto anionic mesoporous silica nanoparticles retains antibacterial activity and enhances biocompatibility. Int. J. Pharm..

[28] Koneru B., Shi Y., Wang Y.-C., Chavala S.H., Miller M.L., Holbert B., Conson M., Ni A., Di Pasqua A.J. (2015). Tetracycline-containing MCM-41 mesoporous silica nanoparticles for the treatment of Escherichia coli. Molecules.

[29] Colilla M., Vallet-Regí M. (2020). Targeted stimuli-responsive mesoporous silica nanoparticles for bacterial infection treatment. Int. J. Mol. Sci..

[30] Rădulescu D., Voicu G., Oprea A.E., Andronescu E., Grumezescu V., Holban A.M., Vasile B.S., Surdu A.V., Grumezescu A.M., Socol G. (2016). Mesoporous silica coatings for cephalosporin active release at the bone-implant interface. Appl. Surf. Sci..

[31] Chen Y., Chen H., Zhang S., Chen F., Zhang L., Zhang J., Zhu M., Wu H., Guo L., Feng J. (2011). Multifunctional mesoporous nanoellipsoids for biological bimodal imaging and magnetically targeted delivery of anticancer drugs. Adv. Funct. Mater..

[32] He Q., Zhang J., Shi J., Zhu Z., Zhang L., Bu W., Guo L., Chen Y. (2010). The effect of PEGylation of mesoporous silica nanoparticles on nonspecific binding of serum proteins and cellular responses. Biomaterials.

[33] Xi G., Liu W., Chen M., Li Q., Hao X., Wang M., Yang X., Feng Y., He H., Shi C. (2019). Polysaccharide-based lotus seedpod surface-like porous microsphere with precise and controllable micromorphology for ultrarapid hemostasis. ACS Appl. Mater. Interfaces.

[34] Gessner A., Lieske A., Paulke B.R., Müller R.H. (2003). Functional groups on polystyrene model nanoparticles: Influence on protein adsorption. J. Biomed. Mater. Res. Part A.

[35] Cristian R.E., Mohammad I.J., Mernea M., Sbarcea B.G., Trica B., Stan M.S., Dinischiotu A. (2019). Analyzing the interaction between two different types of nanoparticles and serum albumin. Materials.

[36] Shegokar R., Al Shaal L., Mitri K. (2011). Present status of nanoparticle research for treatment of tuberculosis. J. Pharm. Pharm. Sci..

[37] (2009). Biological Evaluation of Medical Devices—Part 5: Tests For In Vitro Cytotoxicity. International.

[38] Murashov V., Harper M., Demchuk E. (2006). Impact of silanol surface density on the toxicity of silica aerosols measured by erythrocyte haemolysis. J. Occup. Environ. Hyg..

[39] Son Y.-H., Choy Y.B., Choi H.R., Kim D.S., Park K.C., Choy J.-H. (2007). One-pot synthetic route to polymer–silica assembled capsule encased with nonionic drug molecule. Chem. Commun..

[40] Lu J., Liong M., Zink J.I., Tamanoi F. (2007). Mesoporous silica nanoparticles as a delivery system for hydrophobic anticancer drugs. Small.

[41] Menon N., Leong D.T. (2016). Cytotoxic effects of phosphonate-functionalized mesoporous silica nanoparticles. ACS Appl. Mater. Interfaces.

[42] Slowing I.I., Wu C.W., Vivero-Escoto J.L., Lin V.S.Y. (2009). Mesoporous silica nanoparticles for reducing hemolytic activity towards mammalian red blood cells. Small.

